# Human macrophages limit oxidation products in low density lipoprotein

**DOI:** 10.1186/1476-511X-4-6

**Published:** 2005-03-04

**Authors:** Lillemor Mattsson Hultén, Christina Ullström, Alexandra Krettek, David van Reyk, Stefan L Marklund, Claes Dahlgren, Olov Wiklund

**Affiliations:** 1 Wallenberg Laboratory for Cardiovascular Research, Sahlgrenska University Hospital, SE-413 45 Göteborg, Sweden; 2 Department of Health Sciences, University of Technology, Sydney, N.S.W. 2007, Australia; 3 Medical Biosciences, Clinical Chemistry, Umeå University Hospital, SE-901 85 Umeå, Sweden; 4 Phagocyte Research Laboratory, Department of Rheumatology and Inflammation Research, University of Göteborg, SE-413 46 Göteborg, Sweden

**Keywords:** Macrophages, LDL, lipid peroxides, antioxidant enzymes

## Abstract

This study tested the hypothesis that human macrophages have the ability to modify oxidation products in LDL and oxidized LDL (oxLDL) via a cellular antioxidant defence system. While many studies have focused on macrophage LDL oxidation in atherosclerosis development, less attention has been given to the cellular antioxidant capacity of these cells.

Compared to cell-free controls (6.2 ± 0.7 nmol/mg LDL), macrophages reduced TBARS to 4.42 ± 0.4 nmol/mg LDL after 24 h incubation with LDL (P = 0.022). After 2 h incubation with oxLDL, TBARS were 3.69 ± 0.5 nmol/mg LDL in cell-free media, and 2.48 ± 0.9 nmol/mg LDL in the presence of macrophages (P = 0.034). A reduction of lipid peroxides in LDL (33.7 ± 6.6 nmol/mg LDL) was found in the presence of cells after 24 h compared to cell-free incubation (105.0 ± 14.1 nmol/mg LDL) (P = 0.005). The levels of lipid peroxides in oxLDL were 137.9 ± 59.9 nmol/mg LDL and in cell-free media 242 ± 60.0 nmol/mg LDL (P = 0.012). Similar results were obtained for hydrogen peroxide. Reactive oxygen species were detected in LDL, acetylated LDL, and oxLDL by isoluminol-enhanced chemiluminescence (CL). Interestingly, oxLDL alone gives a high CL signal. Macrophages reduced the CL response in oxLDL by 45% (P = 0.0016). The increased levels of glutathione in oxLDL-treated macrophages were accompanied by enhanced catalase and glutathione peroxidase activities.

Our results suggest that macrophages respond to oxidative stress by endogenous antioxidant activity, which is sufficient to decrease reactive oxygen species both in LDL and oxLDL. This may suggest that the antioxidant activity is insufficient during atherosclerosis development. Thus, macrophages may play a dual role in atherogenesis, i.e. both by promoting and limiting LDL-oxidation.

## Introduction

Oxidative modification of low density lipoprotein (LDL) plays a major role in the pathogenesis of atherosclerosis. The first stage of atherogenesis is characterized by an influx and accumulation of LDL in the intima, followed by recruitment of blood-derived monocytes and lymphocytes to the developing lesion [1]. Subsequently, LDL is oxidatively modified by free radicals that are either secreted from cells within lesions or generated extracellular in the arterial wall [2]. Oxidatively modified LDL (oxLDL) induces a multitude of cellular responses which lead to vascular dysfunction [3]. Much attention has sofar been devoted to the mechanisms by which cells oxidize LDL, since interventions targeting these mechanisms could prevent or retard the disease process.

However, cells may also provide a protective effect by reducing oxidation products present in LDL and oxLDL. Murine macrophages effectively block LDL oxidation by mechanisms which include metal ion sequestration [4]. Recent studies show that macrophages decrease cholesteryl ester hydroperoxide levels in LDL, an antioxidant action that is proportional to cell number [5,6]. In addition, endothelial cells prevent accumulation of lipid hydroperoxides in LDL [7]. Human hepatic cells show a protective role by selective uptake and detoxification of cholesterol ester hydroperoxides present in high density lipoprotein [8]. Enzymes associated with antioxidant defense, such as manganese superoxide dismutases, catalase, and glutathione peroxidases are induced by oxidants in vitro [9-11].

Four selenium-dependent glutathione peroxidases (GPx) have been identified sofar: cytosolic GPx (cGPx), gastrointestinal GPx (GI-GPx), plasma GPx (pGPx), and phospholipid hydroperoxide GPx (PHGPx) [12,13]. The PHGPx reduces hydroperoxides present in complex lipids such as phospholipids and cholesteryl esters [14,15]. Interestingly, increased glutathione levels are present in macrophages derived from the human monocytic cell line THP-1, as well as in mouse peritoneal macrophages after incubation with oxLDL [16,17]. An increased activity of both glutathione peroxidase and superoxide dismutase occurs in the arterial wall of cholesterol-fed rabbits [18]. Furthermore, lipid-laden macrophages within atherosclerotic vessels express an extracellular form of superoxide dismutase (EC-SOD) [19].

This study tested the hypothesis that human macrophages have the ability to modify oxidation products in LDL and oxLDL. We also analyzed the activity of cellular antioxidant defenses such as catalase, glutathione peroxidase, and superoxide dismutase in these cells. We used early macrophages as cell culture model to mimic newly recruited macrophages into the intima.

## Materials and methods

### Cell isolation

Mononuclear cells were isolated by the Ficoll-Hypaque procedure (Pharmacia, Uppsala, Sweden) [20] from buffy coats obtained from the blood of healthy donors from the Blood Bank at Sahlgrenska University Hospital, Göteborg. Monocytes in RPMI 1640 medium (Life Technologies, Paisley, Scotland), supplemented with non-essential amino acids, 2 mM sodium pyruvate, 100 U/mL penicillin, and 100 μg/mL streptomycin were seeded in 6 well plates at 4 × 10^6 ^cells per well. Non-adherent cells were removed after 1 h. RPMI 1640 containing 100 μg/mL LDL or oxLDL was incubated at 37°C in 5% CO_2 _in the presence or absence of macrophages. By definition, monocytes are denoted macrophages when they are attached, thus the cells used in this study are considered early human monocyte-derived macrophages (HMDM).

For chemiluminescence experiments, monocytes were allowed to adhere to cell culture flasks for 1 h. Adhered macrophages were then detached by incubation with PBS containing 5 mM EDTA and 2% fetal calf serum for 20 minutes at +4°C [21]. Cells were collected, washed, and resuspended to a density of 5 × 10^6 ^cells/mL in Krebs-Ringer Bicarbonate buffer supplemented with glucose (KRG) (Sigma, St. Louis, Missouri). To obtain non-viable macrophages, cells were stored at +4°C for 16 h. Trypan blue exclusion test confirmed that 100% of the cells were non-viable.

### Lipoproteins

Fresh human EDTA-plasma was obtained from healthy male donors after overnight fasting. LDL (density 1.019–1.063 g/L) was isolated by sequential ultracentrifugation [22]. Before oxidation, native LDL was desalted on a PD-10 column equilibrated with PBS containing 100 μg/mL penicillin and 100 μg/ml streptomycin (PEST) using PBS-PEST as elution buffer. The LDL was oxidized at 37°C for 2–24 hours by 12.5 μmol CuSO_4_/mg LDL. Oxidation was terminated through the addition of 0.5 mmol/L EDTA. The oxLDL was purified on a PD-10 column with PBS as elution buffer and sterilized by filtration through a 0.22 μm filter. Native LDL was acetylated as described [23]. Oxidation of LDL was determined as the relative electrophoretic mobility (REM), i.e. the ratio between the distance oxLDL and native LDL migrate on a 0.5% agarose gel. The LDL in this study was oxidized for 2 h and had a REM ranging from 1.06 to 1.32 and TBARS values between 3 and 8 nmol MDA/mg LDL protein. Lipoprotein concentrations were determined with the BioRad protein assay using γ-globulin as standard.

### Chemiluminescence

The chemiluminescence (CL) assay was performed at 37°C and the CL detected for at least 100 minutes with a luminescence counter (Bio Orbit Luminometer 1251, Turku, Finland) [21]. The CL response was detected in a total volume of 1.0 mL, containing 10 μg isoluminol (Sigma), 4 U horseradish peroxidase (Roche AB, Stockholm, Sweden), and 100 μg of either LDL, oxLDL, or acLDL in KRG in the presence or absence of 5 × 10^5 ^cells. As a specific inhibitor of hydrogen peroxide, 30 μg catalase (Roche AB) was added in some experiments to oxLDL without cells.

### Measurement of oxidation products

Thiobarbituric acid-reactive substances (TBARS) were determined by the method of Yagi [24]. Fluorescence was measured at 553 nm with 515 nm excitation. Lipid peroxides (LPO) were determined by the Lipohydrox assay from Wak-Chemie Medical (Bad-Soden, Germany). Lipid peroxides are reduced to hydroxyl derivatives in the presence of hemoglobin, and the chromogen 10-N-methylcarbamyl-3, 7-dimethylamino-10H-phentiazine is oxidatively cleaved to form methylene blue. Lipid peroxides are quantitated by colorimetric measuring of the methylene blue at 675 nm.

Levels of hydrogen peroxide equivalents (H_2_O_2eq_) were analyzed in the LDL-containing media incubated with or without macrophages. The assay is based on the oxidation of ferrous ions to ferric ions by hydrogen peroxide at acidic pH (OXIS International Inc., Portland, Oregon). The ferric ion binds to the indicator dye xylenol-orange to form a stable complex which is measured at 560 nm.

Apolipoprotein B (Apo B) concentration was determined in cell culture media by immunoprecipitation enhanced by polyethylene glycol at 340 nm (Thermo Clinical Labsystems, Espoo, Finland). ApoB analyses were performed on a Konelab 20 autoanalyser (Thermo Clinical Labsystems).

### Analysis of antioxidant properties of macrophages

The intracellular levels of glutathione and the activity of GPx were measured in crude extracts from macrophages incubated with either LDL or oxLDL. The cells were washed twice with ice-cold PBS and harvested in 0.5 mL lysis buffer. For glutathione peroxidase measurements, this buffer contained 50 mM Tris-HCl, pH 7.5, 5 mM EDTA, 1 mM dithiothreitol. For glutathione measurements, the cells were lysed in 0.5 mL ice-cold 5% metaphosphoric acid. The lysate was spun down at 3000 × g, and the supernatants stored at -80°C.

The presence of glutathione peroxidase was determined with a colorimetric assay (Bioxytech GPX-340) and glutathione (GSH) was measured with Bioxytech GSH-400, both from OXIS International Inc. The assay GPX-340 measures the functional activity of the GPx. In functional terms, all four types of GPx (cGPx, GI-GPx, pGPx, and PHGPx) appear similar in catalytic activity [12,25].

ROOH + 2GSH ----^GPx^----> ROH + GSSG(oxidized glutathione)+ H2O

GSSG + NADPH^+ ^+ H^+ ^--------> 2GSH + NADP^+^

Catalase activity in macrophages was quantified by the method of Aebi [26]. Decomposition of H_2_O_2 _was measured in cell lysates at 240 nm. One unit of catalase activity was defined as the rate constant for the reaction using purified catalase (Roche AB) as standard.

In the cell lysates, the CuZn-superoxide dismutase (SOD) and Mn-SOD enzymatic activities were measured with a direct spectrophotometric method [27]. Extracellular SOD protein was determined by ELISA essentially as previously described [28].

### Data analyses

Data were expressed as means ± standard error. Statistical analysis was performed using Student's paired t-test or ANOVA. P values < 0.05 were considered statistically significant.

## Results

### Macrophages diminish oxidation products in LDL and oxLDL

To study the effect of macrophages on LDL oxidation, we measured TBARS, LPO, and H_2_O_2 _in LDL-containing culture media after 2 h and 24 h incubation with or without macrophages.

**Figure 1:**
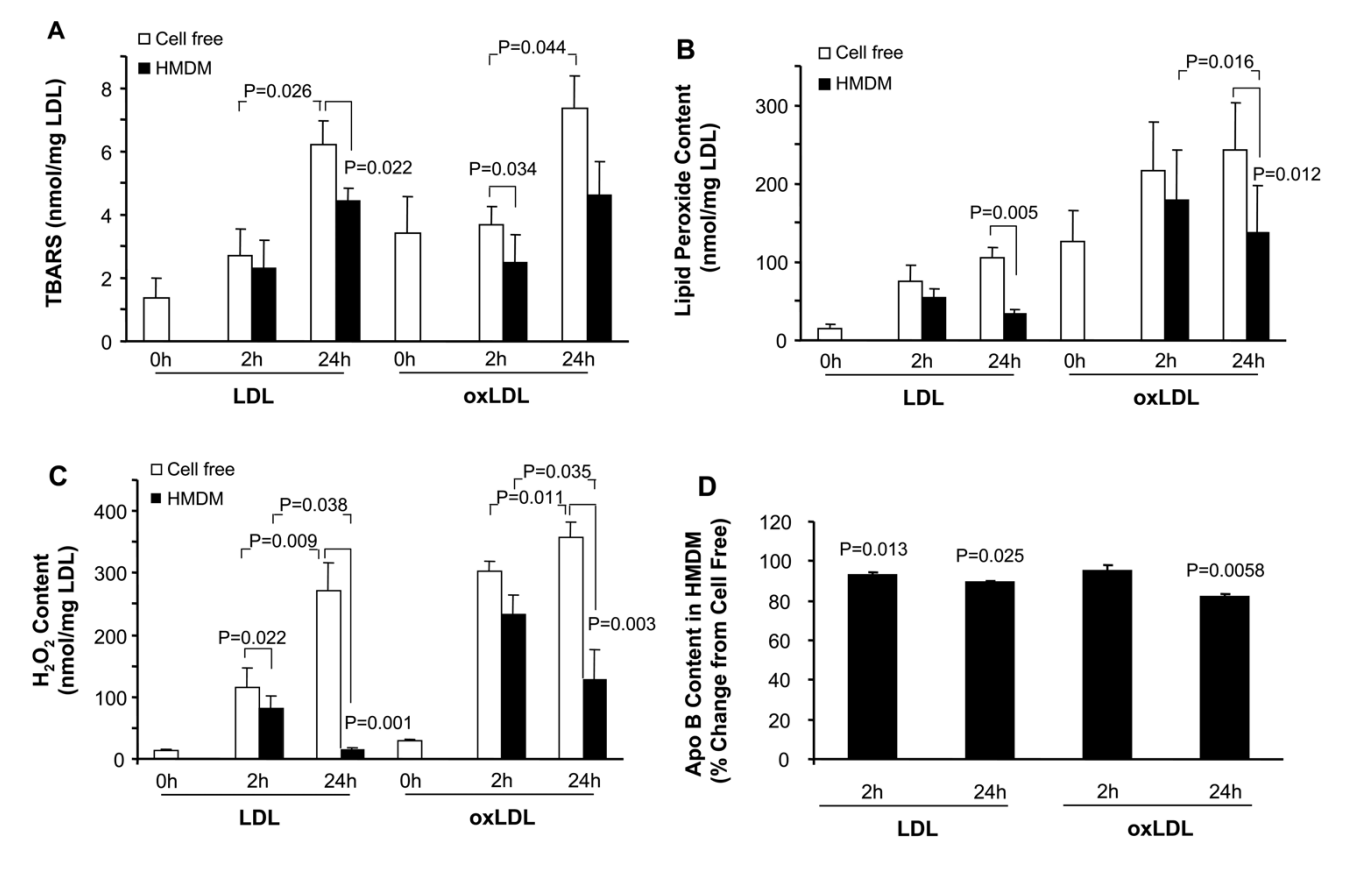
**Effect of macrophages on oxidation products in LDL and oxLDL**. RPMI 1640 containing 100 μg/mL of LDL or oxLDL (oxidized for 2 h) was incubated in cell culture wells at 37°C with and without macrophages (HMDM) for 2 h and 24 h. Values are expressed as nmol/mg LDL, TBARS (n = 5) (A), lipid peroxides (n = 6) (B), H_2_O_2 _(n = 6), (C). Values of Apo B are expressed as % change compared to cell free control incubations (n = 4) (D).

Compared to cell-free controls (6.2 ± 0.7 nmol/mg LDL), there was a significant reduction of TBARS by macrophages to 4.42 ± 0.4 nmol/mg LDL after 24 h incubation with LDL (P = 0.022) (Fig. 1A). After 2 h incubation with oxLDL, TBARS was 3.69 ± 0.5 nmol/mg LDL in cell free media, and 2.48 ± 0.9 nmol/mg LDL in the presence of macrophages (P = 0.034). Although a time-dependent increase of TBARS in the presence of cells is seen, this change was not statistically significant. Lipid peroxide levels are unaffected in LDL and oxLDL incubated in cell-free wells during 2 h to 24 h (Fig. 1B). In the presence of cells, a reduction of lipid peroxides in LDL (33.7 ± 6.6 nmol/mg LDL) was found after 24 h compared to cell-free incubation (105.0 ± 14.1 nmol/mg LDL) (P = 0.005). In oxLDL, the cell-mediated loss of lipid peroxides was significant compared to cell-free media after 24 h. The levels of lipid peroxides in oxLDL were 137.9 ± 59.9 nmol/mg LDL and in cell-free media 242 ± 60.0 nmol/mg LDL (P = 0.012). In LDL-containing media incubated in cell-free wells, the levels of H_2_O_2eq _increase with time from 116 ± 31 nmol/mg LDL after 2 h, to 270 ± 46 nmol/mg LDL after 24 h (P = 0.009) (Fig. 1C). Levels of H_2_O_2eq _increase from 303 ± 14 nmol/mg LDL to 357 ± 24 nmol/mg LDL in oxLDL-containing media (P = 0.011), suggesting both LDL and oxLDL are oxidized during culture conditions. H_2_O_2eq _is significantly decreased in both LDL (P = 0.038) and oxLDL (P = 0.035) after incubation with macrophages. In macrophage-treated LDL, the H_2_O_2eq_content is decreased to 14.8 ± 2.9 nmol/mg LDL after 24 h, which is similar to levels found in non-treated LDL (0 h). Taken together, these results suggest a cell-mediated loss of oxidation products in both LDL and oxLDL in the presence of macrophages.

Apo B was analysed to study if the cell mediated decrease in oxidation products was due to a decrease of LDL or oxLDL in the cell culture media. In the presence of macrophages, the content of apo B in native LDL is reduced by 7 % after 2 h (P = 0.013) and 11 % after 24 h (P = 0.025) (Fig. 1D). In the oxLDL medium, no significant change of apo B in the medium is found after 2 h, and the content of apo B in oxLDL decreases 18% after 24 h incubation with macrophages compared to cell-free control (P = 0.006). This result suggests that the decrease in oxidation products can not be fully explained by an increased uptake of LDL or oxLDL by macrophages.

**Figure 2:**
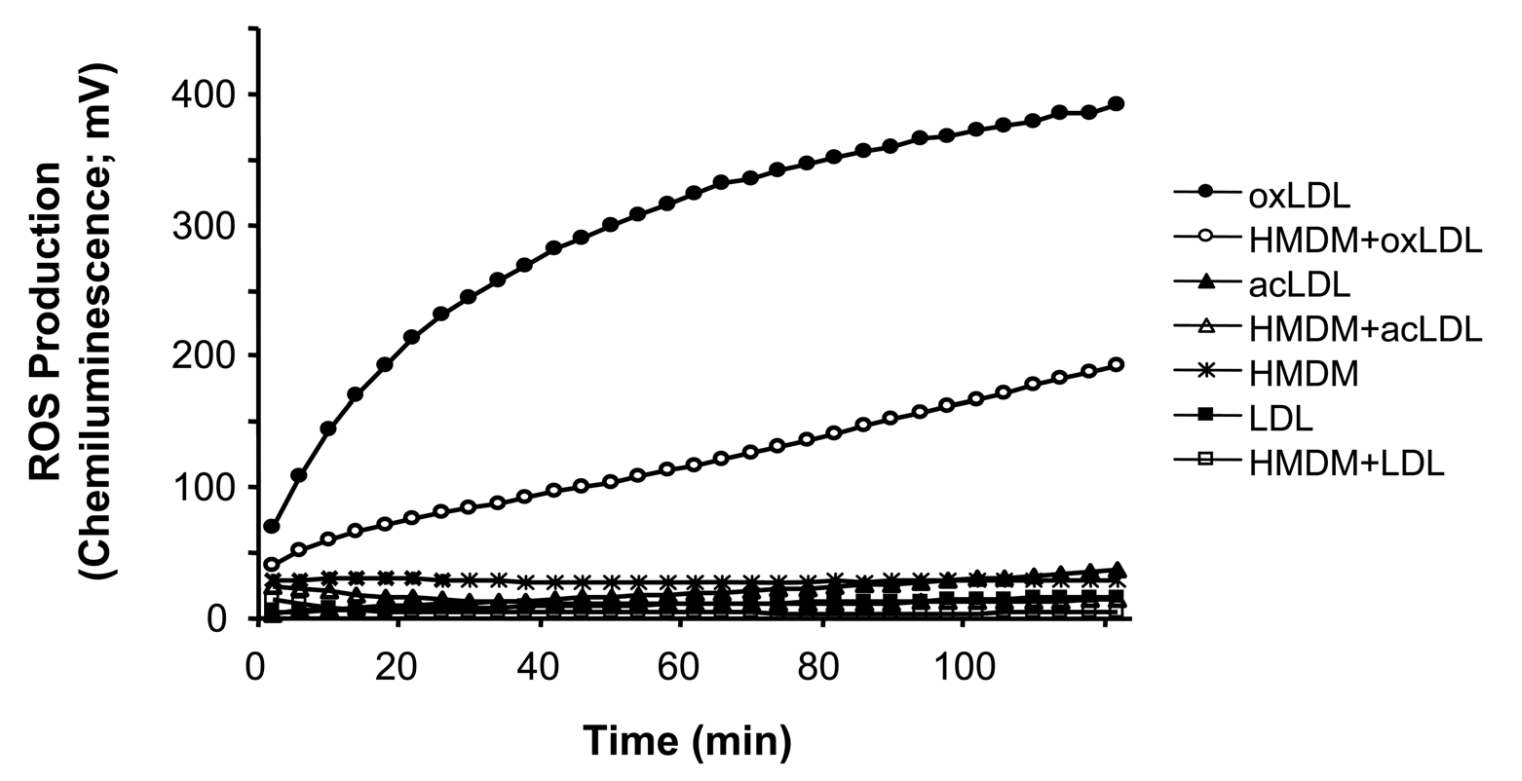
**Production of reactive oxygen species from LDL, oxLDL and macrophages as measured by isoluminol-enhanced chemiluminescence**. The incubation mixture of 1.0 mL KRG contained 5 × 10^5 ^cells (HMDM), 10 μg isoluminol, 4 U horseradish peroxidase, and 100 μg of LDL, oxLDL (oxidized for 2 h), or acLDL. The chemiluminescence was measured every 2 minutes for a total of 100 minutes at 37°C (n = 5).

To further test the capacity of macrophages to decrease oxidation products in LDL, we used isoluminol-enhanced chemiluminescence to detect reactive oxygen species in LDL. OxLDL, acLDL, or LDL was incubated with macrophages (5 × 10^5^cells) or without cells at 37°C. OxLDL alone had a CL response of approximately 400 mV after 2 h (Fig. 2). AcLDL shows a low but increasing CL up to 30 mV after 2 h as does native LDL, but the response is lower than that of oxLDL. In repeated experiments, incubation of macrophages with oxLDL leads to a significant reduction in the maximum peak value to 260 ± 45 mV compared to 462 ± 127 mV for oxLDL alone (P < 0.01) (n = 6). Adding catalase to oxLDL leads to a decrease of the CL signal by 37%, suggesting peroxides in the oxLDL.

**Figure 3:**
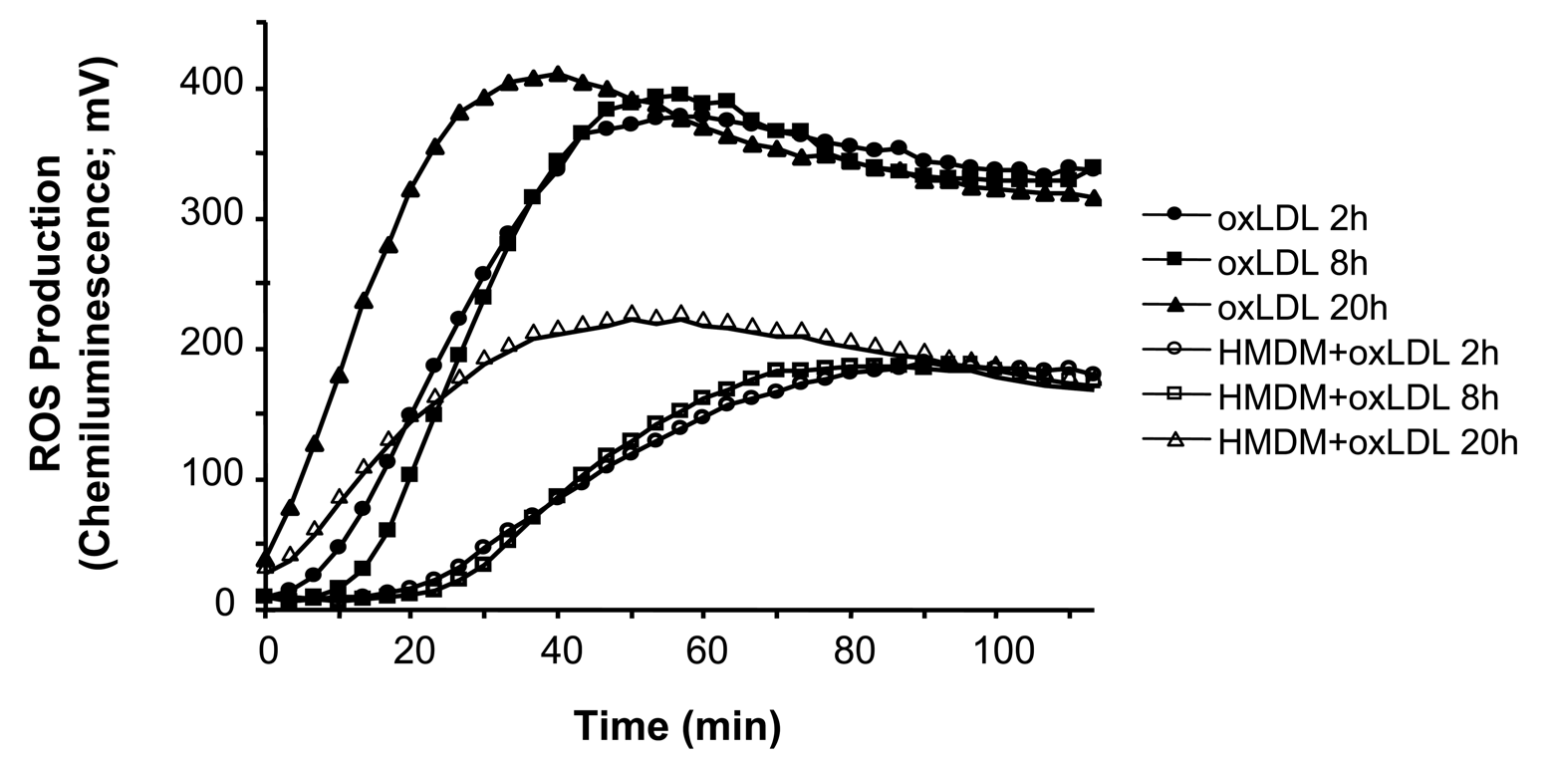
**Production of reactive oxygen species from oxLDL and macrophages as measured by isoluminol-enhanced chemiluminescence**. The incubation mixture of 1.0 mL KRG, containing 10 μg isoluminol, 4 U horseradish peroxidase, and 100 μg of oxLDL (oxidized for either 2 h, 8 h or 20 h), was used alone or in combination with 5 × 10^5 ^macrophages (HMDM) (n = 3).

**Figure 4:**
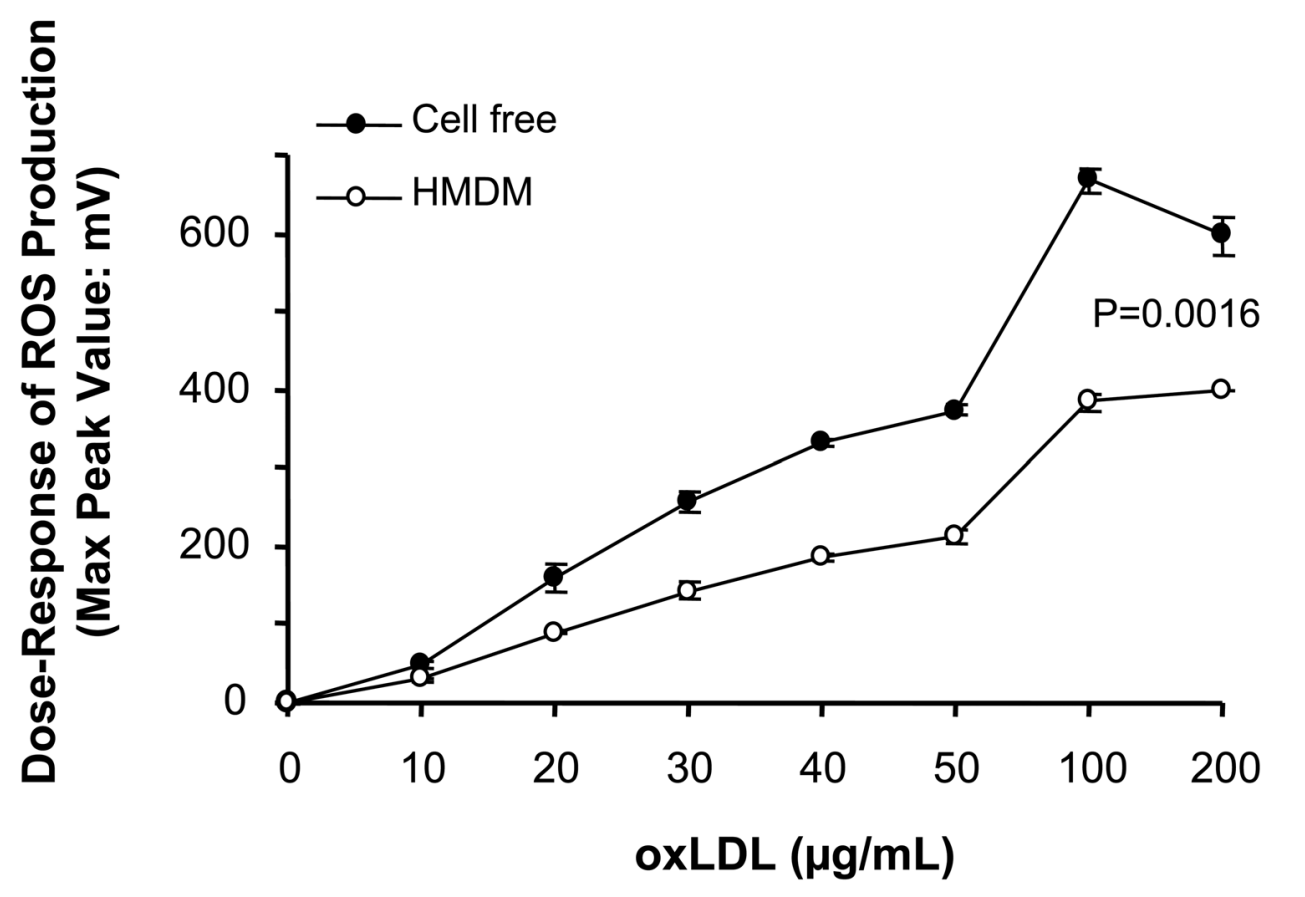
**Dose-response effect of oxLDL on production of reactive oxygen species as measured by isoluminol-enhanced chemiluminescence**. The incubation mixture of 1.0 ml KRG contained 10 μg isoluminol, and different concentrations of oxLDL oxidized for 2 h, alone and in combination with HMDM (5 × 10^5 ^cells). Data are shown as means of maximal peak values in mV ± SE for triplicate determinations within a single experiment and are representative of two independent experiments. Results of oxLDL in cell-free incubations versus oxLDL in the presence of macrophages were analyzed by ANOVA.

LDL oxidized by Cu^2+ ^for 2, 8, or 20 h, shows similar maximum peak values, however a lag time of 10 min is observed with the shorter oxidation times. Incubation of oxLDL with macrophages leads to a reduction in the CL signal (Fig. 3). Different dilutions of oxLDL lead to a dose dependent increase in CL (Fig. 4). The addition of macrophages results in a 45% lower CL maximal peak value at the different concentrations of oxLDL (P = 0.0016).

**Figure 5:**
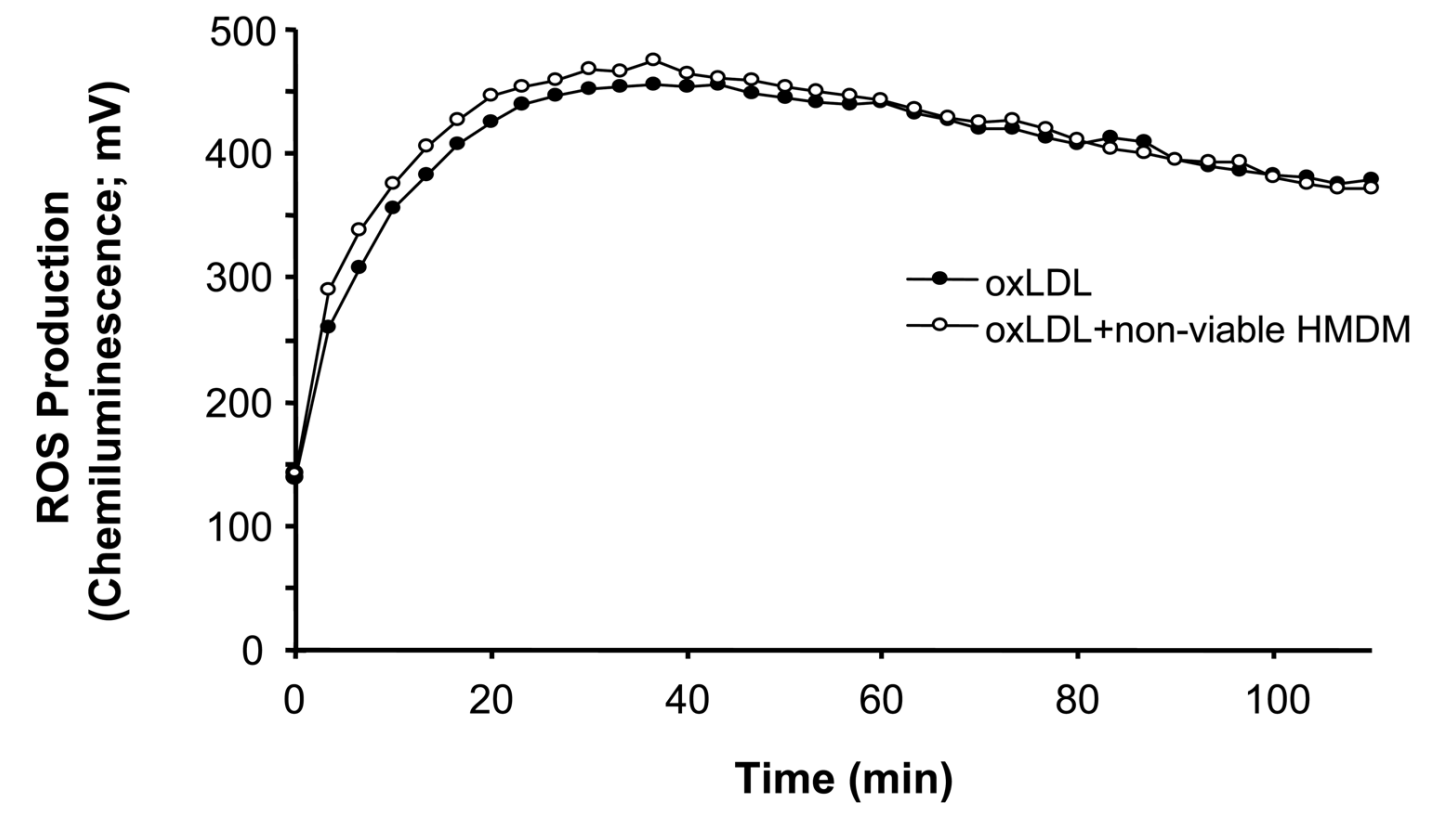
**Production of reactive oxygen species from oxLDL and non-viable macrophages as measured by isoluminol-enhanced chemiluminescence**. The incubation mixture of 1.0 mL KRG, containing 10 μg isoluminol, 4 U horseradish peroxidase, and 100 μg of oxLDL (oxidized for 2 h), was used alone or in combination with 5 × 10^5 ^non-viable macrophages. Data are shown from a typical experiment with 2 different cell donors.

To exclude the possibility that quenching contributes to the macrophage effect, oxLDL was incubated with non-viable macrophages. No reduction in CL signal is seen (Fig. 5), which suggests that no quenching occurred and that viable cells are necessary for antioxidative activity.

### LDL affects cellular antioxidant defences in macrophages

**Figure 6:**
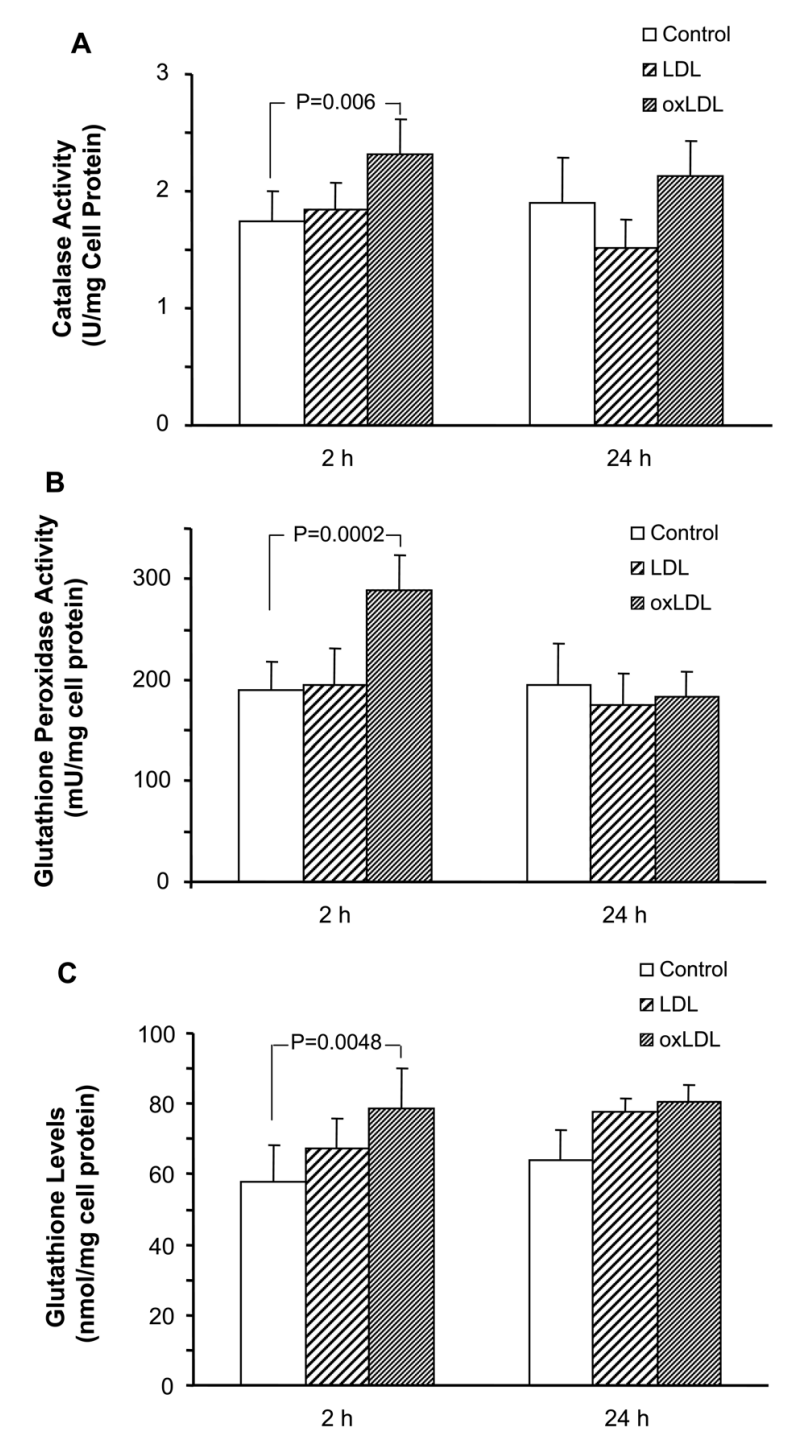
**Effect of LDL and oxLDL on the intracellular antioxidant defenses in macrophages**. The intracellular activity of catalase (A), glutathione peroxidase (B), and the levels of glutathione (C) were measured in crude extracts from macrophages (n = 6) incubated with LDL or oxLDL (oxidized for 2 h). Control cells were incubated in the absence of LDL. Results were analyzed by ANOVA.

Since the levels of peroxides are elevated in oxLDL compared to LDL, and the cellular defences against peroxides excess are catalase and GPx, we investigated the cellular activity of these enzymes in macrophages. Macrophages incubated with oxLDL for 2 h have increased intracellular activity of catalase (P = 0.006) and GPx (P = 0.0002) (Figs 6A and 6B). In contrast, no significant increase of the intracellular activity of these enzymes occurs in macrophages incubated with LDL. In addition, the expression of glutathione, which is a cofactor for the GPx enzymes when H_2_O_2 _is detoxified, is enhanced in macrophages treated with oxLDL for 2 h (P = 0.0048) (Fig. 6C).

Neither LDL nor oxLDL significantly affect the CuZn-SOD or Mn-SOD activity in macrophages (Table I). However, the Mn-SOD activity is enhanced after 24 h compared to 2 h incubations. Although an increase in secreted EC-SOD is seen at 24 h, this change was not statistically significant. Neither LDL nor oxLDL affect this secretion. These observations suggest that macrophages respond to oxLDL by increasing their enzyme activity of catalase and glutathione peroxidase.

**Table 1:** Effect of LDL and oxLDL on superoxide dismutase isoenzymes in macrophages.

	CuZnSOD U/mg cell protein	MnSOD U/mg cell protein	EC-SOD secreted ng/mg cell protein
Control 2 h	81.1 ± 7.5	8.1 ± 0.7	0.61 ± 0.28
LDL 2 h	70.9 ± 8.1	8.2 ± 0.7	0.59 ± 0.23
oxLDL 2 h	71.8 ± 9.0	7.4 ± 0.4	0.45 ± 0.15
			
Control 24 h	79.3 ± 12.1	18.6 ± 1.8 (P = 0.0016)	0.86 ± 0.35
LDL 24 h	80.8 ± 9.9	15.4 ± 0.7 (P = 0.0004)	0.85 ± 0.31
oxLDL 24 h	91.2 ± 20.6	17.1 ± 1.9 (P = 0.0025)	0.80 ± 0.30

Values are means ± SE (n = 4). CuZn-SOD and Mn-SOD enzymatic activities were measured in cell lysates, and EC-SOD protein was determined in culture media. P values indicate the comparison between the effect of LDL/oxLDL on superoxide dismutase at 2 h and 24 h.

## Discussion

Oxidation of LDL is a crucial event in the pathophysiology of atherosclerosis. Reactive oxygen species such as H_2_O_2 _participate in the oxidation of LDL [29]. Although the mechanisms are not fully understood, aortic cells such as endothelial cells, smooth muscle cells, and macrophages have the capacity to oxidize LDL *in vitro*. We have recently shown that hypoxia enhances both macrophage-mediated LDL oxidation and the expression of the putative LDL-oxidizing enzyme 15-lipoxygenase-2 [30]. While many studies have focused on cellular oxidation of LDL, less attention has been given to the cellular antioxidant capacity of macrophages.

This study shows that macrophages play an important role in limiting lipid oxidation products that accumulate in LDL and oxLDL. Since early macrophages are used in this study, this may resemble newly recruited macrophages entering into the arterial intima. Regarding oxLDL, macrophages decrease TBARS levels by about 30%, LPO decreases 43%, and the H_2_O_2eq_content decreases 64% in cell culture media after 24 h compared to cell-free controls. These results are in agreement with an earlier study where human macrophages reduce the content of cholesteryl ester hydroperoxides in LDL by 43% [5]. When the apo B content was analyzed in the culture media, we found that the levels of apo B in oxLDL were 18% reduced after 24 h incubation with macrophages compared to cell free control. For LDL the corresponding figure was 11%, which implies that the decrease in oxidation products cannot be entirely explained by a higher uptake of LDL or oxLDL by macrophages. Our results suggest that macrophages respond to oxidative stress by an endogenous antioxidative activity, which is sufficient to decrease reactive oxygen species; i.e. TBARS, LPO and H_2_O_2 _both in LDL and oxLDL. Our data also suggest that oxidation products accumulate in LDL and in oxLDL during regular cell culture conditions in the absence of cells. This has to be taken into consideration when cell culture data using LDL are interpreted.

The chemiluminescence technique is generally used to detect cellular ROS production. The amplifying molecule isoluminol reacts with ROS to produce an excited state intermediate that emits light upon relaxation to the ground state [31]. Our data show that oxLDL contains reactive oxygen metabolites that have the capacity to induce CL. Lipid hydroperoxides, the major oxidizing species in oxLDL, are likely to cause the CL. In the presence of transition metal ions, they generate Fenton-type oxidants, which may induce the chemiluminescence. The CL response is reduced when LDL is oxidized longer than 24 h. This agrees with previous data concerning the kinetics of LDL oxidation where lipid hydroperoxides are formed during the propagation phase and are decreased during the decomposition phase [32]. In the presence of macrophages, the CL response in oxLDL is reduced by 45%, which suggests that these cells exhibit antioxidant activity. Our results further suggest that cellular viability is necessary for antioxidant activity.

There are a number of cellular defences against oxidative stress, such as superoxide dismutase, catalase, and glutathione-related enzymes. In this study we sought to define the antioxidative activity of macrophages. All glutathione peroxidases reduce H_2_O_2 _or soluble alkyl peroxides, by coupling its reduction of H_2_O with oxidation of glutathione [11]. We found increased glutathione peroxidase activity that coincided with enhanced glutathione levels in oxLDL-treated macrophages. Only PHGPx reduces hydroperoxy groups of lipids together with those of phospholipids and cholesteryl esters when present in lipoproteins [15]. Interestingly, overexpression of PHGPx inhibits H_2_O_2_-induced oxidation and activation of NFκB in transfected rabbit smooth muscle cells [33]. Our results may have *in vivo *relevance, since an increased activity of glutathione peroxidase is found in the artery wall of cholesterol-fed rabbits [18].

Catalase is a representative antioxidant enzyme and previous studies show that oxidants such as H_2_O_2 _and lipid peroxides induce catalase gene expression in cultured rabbit endothelial cells, rabbit macrophages, and human smooth muscle cells [10]. This study provides further evidence that lipid hydroperoxides in oxLDL induce antioxidant defences in macrophages, since human macrophages also upregulated their catalase activity. Human macrophages induce catalase activity in response to oxidative stress [34]. Addition of catalase to oxLDL alone reduced the CL response by 37%, suggesting that peroxides are detected by the CL technique.

EC-SOD occurs in high concentration in both non-diseased [35] and atherosclerotic arterial walls [19]. In non-diseased arteries, the enzyme is primarily secreted by smooth muscle cells, whereas in atherosclerotic lesions it is also expressed by macrophages [19]. Higher levels of EC-SOD are present in cell culture media after 24 h than after 2 h, but the presence of LDL or oxLDL does not effect EC-SOD expression. Similar results of EC-SOD expression have been described in human fibroblasts [36]. The expression of CuZn-SOD is neither affected by LDL, oxLDL nor time. This was not unexpected since CuZn-SOD is generally regarded as a constitutively expressed enzyme. Mn-SOD activity increases with time, but there is no additive effect of LDL or oxLDL on its activity.

Cells can tolerate mild oxidative stress, which triggers the antioxidant defence system in an attempt to restore the oxidant- antioxidant balance [37]. We did not find an increase in antioxidant enzyme activity in LDL-treated macrophages, which suggests that the basal levels of catalase and GPx activity are sufficient to remove the oxidation products in LDL. In contrast, the augmented catalase and GPx activity suggests that oxLDL induces cellular adaptation when macrophages are exposed to increased oxidative stress.

This study suggests that oxidative stress induced by oxLDL could be balanced by a cellular antioxidant defence by newly recruited macrophages to sites of LDL oxidation. As oxidation of LDL is implicated in the development of atherosclerosis, and oxidation products of LDL are present in advanced atheromatous lesions, this may suggest that the antioxidant activity is insufficient *in vivo*. Thus, it is evident that macrophages play a dual role in atherogenesis, i.e. both by promoting and limiting LDL-oxidation. It remains to be determined during which stage of lesion development these individual characteristics pertain.

A strategy to intervene with the development of atherosclerosis would be to increase the endogenous intracellular antioxidant capacity of cells, which would remove and detoxify oxidized LDL. Interestingly, recent results show that Lovastatin increases hepatic catalase activity in cholesterol-fed rabbits [38]. In light of the response-to-retention hypothesis of atherosclerosis, subendothelial retention of atherogenic LDL is the initiating event of the disease [39,40]. It is tempting to speculate that macrophages are present in the atherosclerotic intima because of their antioxidant activity, which detoxifies and removes cytotoxic products in the retained LDL.

## Authors' contributions

All authors have contributed to the design of the study, the data analysis, and the writing of the manuscript. The final version of the manuscript has been read and approved by all authors prior to submission. Each author's specific contribution was as follows; LMH.: study design, macrophage cell culture, CL analysis, LDL treatment. C.U.: macrophage cell culture, TBARS, LPO, GpX, and catalase analyses. A.K.: data interpretation, writing, and editing. D.v.R: CL analysis. S.L.M: SOD analyses. C.D.: setting up the CL method. O.W.: study coordination and data interpretation.
